# Active Smoking, Passive Smoking, and Breast Cancer Risk: Findings from the Japan Collaborative Cohort Study for Evaluation of Cancer Risk

**DOI:** 10.2188/jea.18.77

**Published:** 2008-04-10

**Authors:** Yingsong Lin, Shogo Kikuchi, Koji Tamakoshi, Kenji Wakai, Takaaki Kondo, Yoshimitsu Niwa, Hiroshi Yatsuya, Kazuko Nishio, Sadao Suzuki, Shinkan Tokudome, Akio Yamamoto, Hideaki Toyoshima, Mitsuru Mori, Akiko Tamakoshi

**Affiliations:** 1 Department of Public Health, Aichi Medical University School of Medicine.; 2 Department of Public Health, Nagoya University Graduate School of Medicine.; 3 Department of Preventive Medicine/Biostatistics and Medical Decision Making, Nagoya University Graduate School of Medicine.; 4 Department of Medical Technology, Nagoya University School of Health Sciences.; 5 Department of Health Promotion and Disease Prevention, Nagoya City University Graduate School of Medicine.; 6 Infectious Diseases Division, Hyogo Prefectural Institute of Public Health and Environmental Sciences.; 7 Department of Public Health, Sapporo Medical University School of Medicine.

**Keywords:** Smoking, Breast Neoplasms, Risk, Cohort Studies

## Abstract

**Background:**

Evidence is lacking regarding the relationship between cigarette smoking and breast cancer in Japanese women. We examined the association between breast cancer incidence and active and passive smoking in the Japan Collaborative Cohort Study for Evaluation of Cancer Risk.

**Methods:**

Our study comprised 34,401 women aged 40-79 years who had not been diagnosed previously with breast cancer and who provided information on smoking status at baseline (1988-1990). The subjects were followed from enrollment until December 31, 2001. Cox proportional-hazards models were used to estimate the hazard ratio (HR) and 95% confidence interval (CI) for the association between breast cancer incidence and tobacco smoke.

**Results:**

During 271,412 person-years of follow-up, we identified 208 incident cases of breast cancer. Active smoking did not increase the risk of breast cancer, with a HR for current smokers of 0.67 (95% CI: 0.32-1.38). Furthermore, an increased risk of breast cancer was not observed in current smokers who smoked a greater number of cigarettes each day. Overall, passive smoking at home or in public spaces was also not associated with an increased risk of breast cancer among nonsmokers. Women who reported passive smoking during childhood had a statistically insignificant increase in risk (HR: 1.24; 95% CI: 0.84-1.85), compared with those who had not been exposed during this time.

**Conclusion:**

Smoking may not be associated with an increased risk of breast cancer in this cohort of Japanese women.

## INTRODUCTION

Breast cancer is a complex, multifactorial disease with a strong interaction between genetic and environmental factors.^1^ The role of cigarette smoking in breast cancer etiology has been investigated extensively over several decades, because it is one of the few potentially modifiable environmental factors that contributes to the development of this malignancy.^2^^,^^3^ However, despite a large number of epidemiologic studies being carried out, it remains controversial whether cigarette smoking is associated with an increased risk of breast cancer. While the majority of studies have shown a weak positive or null association,^4^ the association appears to be strongest in premenopausal women^5^ or in women who started smoking at an early age or smoked before their first full-term pregnancy.^6^ A positive association with passive smoking has also been reported in some studies,^7^^-^^10^ but was not observed consistently in other studies.^11^^-^^14^ A recent review suggested that studies with detailed assessment of exposure to passive smoking were more likely to show a stronger positive association between passive smoking and breast cancer risk.^4^

Only a small number of epidemiologic studies have examined the relationship between smoking and breast cancer in Asian countries,^11^^,^^15^^-^^17^ in which the incidence of breast cancer is generally lower than in Western countries. A recent systematic review, based on studies in Japanese women, concluded that smoking possibly increased the risk of breast cancer in the Japanese population.^18^ However, definitive evidence is still lacking as only one of the three cohort studies included in that review found a positive association in premenopausal women.

The sustained increase in breast cancer incidence in Japanese women over recent decades has become a major public health concern in Japan.^19^ Clarifying the relationship between smoking and breast cancer may provide insights into strategies aimed at preventing this malignancy. In order to better understand the association between active and passive smoking and breast cancer risk, we analyzed data from a large cohort study of mainly postmenopausal women in Japan.

## METHODS

### Study Population

The Japan Collaborative Cohort Study for Evaluation of Cancer Risk (JACC) is a prospective cohort study designed to evaluate cancer risk associated with lifestyle factors in the Japanese population. A full description of the JACC Study is available elsewhere.^20^^,^^21^ Briefly, the study was established in 1988-1990, with 110,792 people (46,465 men and 64,327 women), aged 40 to 79 years, being enrolled from either the general population or from subjects who had municipal health check-ups in 45 areas throughout Japan. All the participants were followed up for all-cause mortality. In addition to mortality, the incidence of cancer was recorded in subjects living in 24 areas in which cancer registry systems were established.

Of the 64,327 women in the original cohort, 38,593 women lived in the 24 areas where data on incidence were available. We excluded women who reported a previous diagnosis of breast cancer (n=161) and women who gave no information on smoking status in the baseline questionnaire (n=4031), leaving 34,401 women eligible for the present analysis. The average age at enrollment was 58.0 years.

We obtained informed consent by requesting the subjects to sign the cover page of the questionnaire. In some areas, informed consent was obtained at a group level, after the purpose of the study and confidentiality of the data had been explained to community leaders. The Ethics Board at Nagoya University School of Medicine approved the study in 2000.

### Data Collection

At baseline, we used a self-administered questionnaire to obtain information on demographic characteristics, tobacco and alcohol use, physical activity and other lifestyle factors. Questions on active cigarette smoking comprised smoking status (never, past or current), age at which smoking started, the number of cigarettes smoked per day, years of smoking, and smoking cessation. Information on passive smoking was collected in nonsmokers by recording the response to the following three questions. Firstly, “In the past, were you exposed to tobacco smoke at home?” Women who responded that they had been exposed to environmental tobacco smoke (ETS) were also asked to report the frequency of this passive exposure as either sometimes, 1-2 days/week, 3-4 days/week, or almost every day. Secondly, “In the past, were you exposed to tobacco smoke in public spaces?” Women who answered ‘yes’ to this question also reported the frequency of this passive exposure. Thirdly, “Were you exposed to tobacco smoke from family members in your childhood?” The subjects were categorized as having been exposed to passive smoking if they reported ever being exposed to tobacco smoke at home or in public spaces.

Information on other potential risk factors for breast cancer, such as alcohol drinking, age at menarche, parity, age at the birth of her first child, and age at menopause, was also collected in the baseline questionnaire.

### Follow-up and Identification of Breast Cancer Cases

Follow-up was conducted from enrollment until December 31, 2001. During this period, population registries in the municipalities were used to ascertain the residential and vital status of the participants. In Japan, registration of death is required by Family Registration Law and theoretically provides complete mortality data. Breast cancer incidence was confirmed mainly through linkage of records to the population-based cancer registry in each area. To complete the incidence data, we also conducted a systematic review of death certificates and reviewed medical records in local major hospitals in some areas.

During the study period, 2.7 percent of the subjects were lost to follow-up due to having left the study areas. The proportion of cancer cases with death certificate only was 5.3 percent (11 of 208 cases). The mortality to incidence ratio for breast cancer was 0.15 in the cohort covered by cancer registries. This value was lower than that available from population-based cancer registries in Japan (between 0.20 and 0.30).^22^

### Statistical Analysis

For each participant in the cohort, the person-years of follow-up were counted as either the time from enrollment to the diagnosis of breast cancer, death from any cause, or the end of follow-up (December 31, 2001), whichever occurred first. For breast cancer cases ascertained only by death certificates, the person-years of follow-up were calculated from enrollment to death from breast cancer. Women who died of causes other than breast cancer or moved out of the study areas were treated as censored cases. We used Cox proportional-hazards models to estimate the hazard ratio (HR) and 95% confidence interval (CI) for the association between breast cancer incidence and tobacco smoke. The analyses on the association between passive smoking and breast cancer risk were limited to nonsmokers. Potential confounding factors included in the multivariable models were age (continuous), study area (Hokkaido and Tohoku, Kanto, Chubu, Kinki, Chugoku, Kyushu), body mass index (BMI; <20.0, 20.0-24.9, 25.0-29.9, or ≥30.0 kg/m^2^), alcohol consumption (never, past, or current), daily walking (seldom or never, 30, 30-59, 60 minutes), parity (nulliparous or 1, 2 or 3, or ≥4 births), age at the birth of her first child (<22, 22-25, or ≥26 years), menopausal status (premenopausal or postmenopausal), age at menarche (<15, 15-16, or ≥ 17 years), use of sex hormones (yes/no) and family history of breast cancer in a first-degree relative (yes/no). These variables were selected as covariates as they are known to, or alternatively have been suspected of modifying the risk of breast cancer. We also conducted analyses limited to postmenopausal women only, as the mean age of the study cohort was 58 years and 66 percent of the women reported their age at menopause in the baseline questionnaire.

All *P* values were 2-sided, with *P*<0.05 indicating statistical significance. All the analyses were performed with SAS^®^ package version 9.1 (SAS Institute, Inc., Cary, NC, USA).

## RESULTS

During 271,412 person-years of follow-up, we identified 208 incident cases of breast cancer. Table 1 shows the distributions of risk factors for breast cancer grouped according to smoking status. Current, ex-, and nonsmokers represented 1.6%, 5.3%, and 93.1% of the cohort population, respectively. Smokers and nonsmokers differed in their alcohol consumption, age at the birth of her first child, age at menopause, and use of sex hormones. Compared with nonsmokers, current smokers tended to drink more alcohol, had given birth at a younger age, became menopausal at an earlier age, and were more likely to be on hormone therapy. There were no significant differences in the other risk factors between current and past smokers.

**Table 1.** Distribution of risk factors for breast cancer in women in the JACC Study, grouped according to smoking status at baseline (1988-1990).

Risk factors	Smoking status at baseline			P^*^

	Never (n=32023)	Current (n=1814)	Past (n=564)	
Age (year)^**^	57.8	56.4	60.9	<0.01
Body mass index (kg/m^2^)	22.9	22.7	23.3	0.06
Family history of breast cancer (%)	1.4	1.6	1.2	0.46
Alcohol consumption (g/day)	8.1	20.0	13.6	<0.01
Age at menarche (yr)	14.9	14.9	14.9	0.68
Age at the birth of her first child (year)	25.1	24.8	24.7	<0.01
Age at menopause (yr)^***^	48.7	47.9	48.4	<0.01
Use of sex hormones (%)	4.9	7.4	3.1	<0.01

Data presented are means otherwise specified.

^*^: The significance of differences in baseline characteristics between current and nonsmokers was tested using the χ^2^ test or Student’s t test, where appropriate.

^**^: The number of women in each 10-year age group were as follows: 40-49, 8345; 50-59, 10772; 60-69, 10412; 70-79, 4872.

^***^: Sixty six percent of the women reported their age at menopause at baseline.

Table 2 summarizes the risk of breast cancer in relation to current and past smoking. After adjustment for potential confounding factors, the relative risk was slightly increased in ex-smokers (HR: 1.27). However, this association was not statistically significant. Cigarette smoking did not increase the risk of breast cancer incidence, with the HR for current smokers being 0.67 (95% CI: 0.32-1.38). In addition, no increased risk was observed for current smokers who smoked a greater number of cigarettes per day.

**Table 2.** Hazard ratios and 95% confidence intervals for breast cancer in relation to active smoking in the JACC Study.

	Person-years	No. of cases	Hazard ratios (95% confidcence intervals)	

			Age-adjusted	Multivariate^*^
Nonsmokers	338113	196	1.00 (reference)	1.00 (reference)
Ex-smokers	5002	4	1.41 (0.53-3.80)	1.27 (0.46-3.48)
Current smokers	17945	8	0.75 (0.37-1.53)	0.67 (0.32-1.38)
0-10 (cigarettes/day)	9291	5	0.92 (0.38-2.24)	0.84 (0.34-2.06)
≧ 11	7766	3	0.64 (0.21-2.01)	0.55 (0.17-1.75)

^*^: Hazard ratios adjusted for age, area, body mass index, family history of breast cancer, alcohol drinking, daily walking, age at menarche, age at the birth of her first child, menopause status at baseline, number of births, and use of sex hormons.

Women who had missing data on number of cigarettes smoked per day were excluded.

Overall, passive smoking at home or in public spaces was not associated with an increased risk of breast cancer in nonsmokers (Table 3). Passive smoking at home appeared to decrease the risk, although this association was not statistically significant. Women who reported passive smoking during childhood had a statistically insignificant increase in the risk of developing breast cancer (HR: 1.24 ; 95% CI: 0.84-1.85), compared with women not exposed to passive smoking during this time.

**Table 3.** Hazard ratios and 95% confidence intervals for breast cancer in relation to passive smoking among nonsmokers in the JACC Study.

	Person-years	No. of cases	Hazard ratios (95% confidcence intervals)	

			Age-adjusted	Multivariate^*^
Passive smoking at home				
No	93043	63	1.00 (reference)	1.00 (reference)
Yes				
Sometimes	38314	17	0.65 (0.38-1.10)	0.59 (0.33-1.05)
Almost everyday	107269	51	0.68 (0.47-0.99)	0.71 (0.48-1.05)

Passive smoking in public spaces				
No	142414	83	1.00 (reference)	1.00 (reference)
Yes				
Sometimes	73607	37	0.84 (0.57-1.24)	0.77 (0.51-1.15)
Almost everyday	36335	20	0.93 (0.57-1.53)	0.84 (0.51-1.40)

Passive smoking during childhood				
No	79623	37	1.00 (reference)	1.00 (reference)
Yes	255172	141	1.21 (0.84-1.74)	1.24 (0.84-1.85)

^*^: Hazard ratios adjusted for age, area, body mass index, family history of breast cancer, alcohol drinking, daily walking, age at menarche, age at the birth of her first child, menopausal status at baseline, number of births and use of sex hormones.

Women who had missing data in each category were excluded.

No significant increase in breast cancer incidence in relation to active smoking was observed in the analyses limited to post-menopausal women only, with the HR being 1.20 (95% CI: 0.52-2.80) for current smokers.

We also conducted an analysis using nonsmokers who had not been exposed to passive smoking either at home or in public spaces as a reference group. The risk estimates for current and past smoking in this group were essentially unchanged (data not shown).

## DISCUSSION

We examined the relationship between cigarette smoking and breast cancer in a large Japanese cohort comprised mainly of postmenopausal women. Our findings indicated that neither active nor passive smoking were associated with an increased risk of breast cancer in our study cohort.

We found no indication of an increased risk for breast cancer in current smokers. Previous studies have provided conflicting results on the association between active smoking and breast cancer risk, with the majority of studies showing no significant association, or only a modest, positive association.^2^^,^^3^ The Nurses’ Health Study concluded that smoking did not increase the risk of breast cancer.^14^ When adjusted for alcohol drinking, no association was observed between cigarette smoking and breast cancer risk in a collaborative reanalysis of individual data from 53 epidemiologic studies.^3^ In contrast, a pooled analysis including both case-control and cohort studies published before December 2004 reported an approximately 40 percent increase in the risk of breast cancer in active tobacco smokers.^4^ However, cohort studies are more likely to show no association, or to yield modest risk estimates compared with case-control studies. This suggests that recall bias or selection bias inherent in case-control studies may influence the association between breast cancer and active smoking.

We have interpreted our finding of a lack of association between active smoking and breast cancer risk from both a biological and methodological perspective. Firstly, it has been suggested that considerable heterogeneity exists in the etiology of breast cancer.^23^ Breast cancer can be classified according to estrogen receptor and/or progesterone receptor expression, with each type having different clinical, pathological and molecular features.^24^ The positive association between breast cancer and smoking may be diluted or masked in studies that treat breast cancer as a single outcome, as we did in our analyses. Secondly, given that the majority of the women in our cohort were nonsmokers at baseline and only the small number of current smokers developed breast cancer during follow-up, it was difficult to detect a statistically significant relationship between smoking and breast cancer risk. Thirdly, concern has been raised previously that studies showing no significant positive associations included many women in the reference group who had been exposed to passive smoking but who had never smoked.^4^ This may explain, in part, the lack of association we observed in our study. However, even when we included women who had never been exposed to either active or passive smoking as the reference group, we observed no increased risk of breast cancer in current smokers. Fourthly, the majority of the women in our cohort were postmenopausal. We therefore conducted an analysis limited to postmenopausal women only and found no significant associations. Accordingly, we consider that our finding of a lack of a significant association between active smoking and breast cancer risk is applicable to postmenopausal Japanese women. However, the lack of information on menopausal status at diagnosis of breast cancer did not allow us to carry out a detailed analysis. Our result is consistent with that of another large cohort study conducted in Japan,^16^ in which no significant association was obtained between smoking and breast cancer incidence in postmenopausal women. However, the possibility remains that smoking may be associated with an increased risk of breast cancer in premenopausal women, as that cohort study showed a 3.9-fold increase in breast cancer risk for premenopausal Japanese women.^16^ In contrast to the inconsistent results for premenopausal women, the majority of studies have shown that cigarette smoking was not associated with an increased risk of breast cancer in postmenopausal women.^3^^,^^4^

In the present study, passive smoking at home or in public places was not associated with an increased risk of breast cancer in nonsmokers. Evidence supporting a positive association between passive smoking and breast cancer risk has been derived mainly from case-control studies.^4^ However, three large cohort studies conducted in the United States did not confirm the positive findings of these case-control studies.^12^^-^^14^ In the Nurses’ Health Study, passive smoking was unrelated to breast cancer risk.^14^ Similarly, a large, prospective study reported no association between ETS from a smoking spouse or from other sources and the risk of death from breast cancer.^12^ In contrast, a recent Japanese cohort study reported a significant 2.6-fold increased risk of breast cancer for passive smoking in premenopausal women.^16^ Moreover, a review by the California Environmental Protection Agency concluded that regular ETS exposure was related to breast cancer diagnosed in younger women who were primarily premenopausal.^25^ The interpretation of these contradictory results is difficult, given the heterogeneity of breast cancer etiology, variation in study design and the precision of ETS measurement. However, active smoking is not an established risk factor for breast cancer, and the amount of exposure is lower from ETS than from active smoking. In addition, previous studies that examined this association were of small sample size and retrospective. On the basis of these facts, we consider it unlikely that passive smoking plays a major role in the development of breast cancer, although it may be related to breast cancer in certain high-risk populations.

The strength of our study was its prospective design, which avoided recall bias inherent in case-control studies. Information on other risk factors for breast cancer was available and potential confounding factors were controlled for in the analyses examining the association between smoking and breast cancer.

The limitations of our study merit consideration. As mentioned earlier, due to the small number of breast cancer cases who were current smokers, we were unable to conduct a detailed analysis examining the association between breast cancer and factors such as age when smoking was started, frequency, and duration. Previous studies suggested that early childhood may be a critical period of risk, and that there may be an increased risk with starting smoking at an earlier age and long duration of smoking.^26^ Another limitation of our study was that we did not collect information on menopausal status at diagnosis of breast cancer, hormonal receptor status or genetic polymorphisms, all of which can be used to define breast cancer risk more accurately.^27^^,^^28^ Finally, the validity of our findings on passive smoking may be a concern given the relatively high percentage of missing data (24.8%) regarding this exposure. Random errors are more likely in this situation and therefore the real association may have been underestimated. Further improvements in the measurement of passive smoking are therefore needed in order to provide more accurate risk estimates for passive smoking and breast cancer.

In conclusion, our results suggest that smoking is not associated with the risk of breast cancer in this cohort of Japanese women.
